# Update on the pathogenesis of vitiligo^☆^

**DOI:** 10.1016/j.abd.2021.09.008

**Published:** 2022-05-25

**Authors:** Helena Zenedin Marchioro, Caio César Silva de Castro, Vinicius Medeiros Fava, Paula Hitomi Sakiyama, Gerson Dellatorre, Hélio Amante Miot

**Affiliations:** a Medical Residency in Dermatology, Hospital Irmandade Santa Casa de Misericórdia de Curitiba, Curitiba, PR, Brazil; b Escola de Medicina, Pontifícia Universidade Católica do Paraná, Curitiba, PR, Brazil; c The Research Institute of the Mcgill University Health Centre, Montreal, QC, Canada; d Universidade Estadual do Oeste do Paraná, Cascavel, PR, Brazil; e Department of Dermatology, Faculty of Medicine, Universidade Estadual Paulista, Botucatu, SP, Brazil

**Keywords:** Autoimmunity, Oxidative stress, Pigmentation, Vitiligo

## Abstract

Vitiligo is a complex disease whose pathogenesis results from the interaction of genetic components, metabolic factors linked to cellular oxidative stress, melanocyte adhesion to the epithelium, and immunity (innate and adaptive), which culminate in aggression against melanocytes. In vitiligo, melanocytes are more sensitive to oxidative damage, leading to the increased expression of proinflammatory proteins such as HSP70. The lower expression of epithelial adhesion molecules, such as DDR1 and E-cadherin, facilitates damage to melanocytes and exposure of antigens that favor autoimmunity. Activation of the type 1-IFN pathway perpetuates the direct action of CD8+ cells against melanocytes, facilitated by regulatory T-cell dysfunction. The identification of several genes involved in these processes sets the stage for disease development and maintenance. However, the relationship of vitiligo with environmental factors, psychological stress, comorbidities, and the elements that define individual susceptibility to the disease are a challenge to the integration of theories related to its pathogenesis.

## Introduction

Vitiligo is a chronic, acquired dyschromia that promotes autoimmune aggression against melanocytes, resulting in hypochromic or achromic macules and patches on the skin and mucous membranes, with possible involvement of hair follicles, in different extensions of the skin, which may accompany systemic manifestations (e.g., sensorineural deafness, uveitis, thyroiditis). Its pathogenesis is multifactorial; however, the exact mechanisms that integrate the individual genetic susceptibility, melanocyte auto aggression, and failure of immune tolerance mechanisms are still not fully elucidated.

The prevalence of vitiligo is quite variable around the world, being more frequent in Africa (0.4%), Europe (0.4%), and Oceania (1.2%), than in North America (0.2%) and Asia (0.1%).^1^ In Brazil, its prevalence varies between 0.46%–0.68% of the population, with no discrepancy between the sexes or racial groups. The mean age of disease onset ranges from 20 to 30 years of age, although it can affect children and the elderly. Vitiligo still accounts for 1.4% to 1.9% of dermatological consultations and up to 3.5% of dermatological consultations in children.^2^, ^3^

Although it does not show specific skin symptoms or imply a serious health risk, vitiligo patients are impacted in their quality of life since the disease is associated with a strong stigma that compromises social and professional relationships, self-esteem, and dress codes; with women, adolescents, and patients with psychiatric disorders being the most affected groups.^4^

There is currently no definitive cure for vitiligo; however, several treatments have shown favorable results, achieving some degree of repigmentation in over 80% of cases.^5^ The recent advance in the understanding of its pathogenesis has promoted new therapeutic alternatives, which allude to a more hopeful future for patients.

## Vitiligo genetics

Vitiligo is a complex disease in which the risk attributed to the genetic component is estimated at 75% to 83%, whereas environmental factors would comprehend the remaining 20%. Studies of familial grouping, studies in twins, and segregation analyses characterize it as a multifactorial disease with a polygenic inheritance pattern. Because of this, the individual contribution of each genetic variant to susceptibility is relatively low.^6^, ^7^

### Vitiligo susceptibility mapping by linkage analysis

The mapping of genetic risk factors for vitiligo was performed by linkage analysis followed by positional cloning. Linkage analysis assesses the nonrandom segregation of chromosomal regions among affected individuals in families with vitiligo. Seven loci have been linked to vitiligo susceptibility, of which four were observed in European populations and three in Chinese populations. One locus on chromosome 17p13 was the first genomic region linked to vitiligo associated with autoimmune diseases.^8^ And the positional cloning of the 17p13 region identified the *NLRP1* gene as the likely source of the vitiligo linkage signal.^9^, ^10^

Chromosome 22q12 has been associated with the pathogenesis of vitiligo,^8^, ^11^ with the variants that regulate *XBP1* gene expression being the risk factor indicated in the 22q12 region.^12^ *XBP1* is a transcription factor that regulates the expression of the HLA class II gene, being involved in cellular response to stress and often associated with autoimmune diseases. In Asian and South American individuals, a link between the MHC region on chromosome 6p21 and vitiligo has been observed.^6^, ^11^

HLA class I and II-related genes have been implicated in the pathogenesis of vitiligo.^13^, ^14^ Fine mapping of HLA by imputation identified amino acid alterations at residues 135 and 45‒46 for HLA-DQB1 and HLA-B, respectively, as strong risk factors for vitiligo in the Chinese population.^15^ Moreover, a promoter variant that increases HLA-A*02:01 expression has been associated with common vitiligo.^16^

Three loci, at 1p31.3 – p32.2, 7q21.11, and 8p12, have been linked to vitiligo susceptibility in Europeans and one in Asian populations at 4q13-q21.^17^, ^18^, ^19^ Of these, FOXD3 has been suggested as the causal gene candidate for 1p31.3 – p32.2 and *PDGFRA* for 4q13-q21, while no candidate gene has been suggested for the two remaining loci.

In addition to these, HLA-A*33, HLA-Aw*31, HLA-DR4, HLA-DR7, and HLA-DQB1*0303, among others, have been identified as risk factors for vitiligo in different samples.^20^, ^21^ On the other hand, there are studies that correlate HLA-A*09 and HLA-Aw*19 with a lower risk for the disease.^21^

In Brazil, a study with patients from the southeast region demonstrated an association of HLA-A*02 and HLA-DRB1*07 with susceptibility to vitiligo.^20^ HLA-A*02 is also associated with risk in populations from China, India, Slovakia, and Northern Germany. Similarly, HLA-DRB1*07 has been identified in samples from China, India, Slovakia, Italy, Morocco, Turkey, and Oman.^20^

Additionally, in Brazil, HLA-DQB1*06 has been correlated with susceptibility to vitiligo (common, acrofacial and mixed), while HLA-A*32 has been correlated with the localized form (focal and segmental). However, these findings differ from other descriptions in the literature, suggesting that HLA-DQB1*06 and HLA-A*32 are specifically associated with the Brazilian population.^20^

### Genome-wide association study and vitiligo prediction

GWAS (Genome-Wide Association Study) tests a dense set of variants located throughout the human genome for association with a phenotype of interest using both case controls and families. To date, five GWAS have been performed for vitiligo in European and Asian populations.^22^, ^23^, ^24^, ^25^, ^26^ Together, they have identified more than 50 loci associated with risk of vitiligo. Most of the loci identified by GWAS were detected in Europeans, suggesting a specific ethnicity effect or differences in study design or power. However, seven of the non-MHC loci were associated with vitiligo in subjects of different ethnicities.

The observation of risk effects in independent populations strengthens the global contribution of genes in the pathophysiology of vitiligo. One of these seven multiethnic vitiligo risk factors includes the *PTPN22* gene, which encodes a protein tyrosine phosphatase involved in T-cell signaling. Interestingly, *PTPN22* variants have been associated with several diseases related to the immune system, including rheumatoid arthritis, systemic lupus erythematosus, and Crohn's disease, which characterizes it as a pleiotropic gene for autoimmune diseases.^27^, ^28^

The *IKZF4* gene, associated with vitiligo in European and Chinese individuals, exerts a FOXP3-mediated gene silencing on T regulatory cells (Tregs).^29^ The *FOXP3* gene located on the X chromosome was also a risk factor for vitiligo in several ethnicities.

Combined, these associations highlight the key players in the contribution of T-cells to the pathogenesis of vitiligo. Other candidate loci for vitiligo in several ethnicities include *FASLG*, a member of the TNF superfamily, and GZMB, a protease, both of which point to a role of dysregulated apoptosis in vitiligo. Another significant non-HLA association with vitiligo found in Europeans was observed for the *TYR* gene, which regulates melanin biosynthesis in melanocytes.^26^

The translational aspect of the GWAS findings goes beyond the description of the mechanisms associated with the disease pathogenesis. The cumulative frequency of vitiligo risk alleles can be used to calculate the probability, using a polygenic risk score, that an individual will become a case. Using autosomal risk variants of vitiligo described by GWAS, the predictive power of the polygenic risk score was found to be 71%, which is among the highest values ​​found for complex diseases.^30^

Candidate gene approaches are conceptually based on a mechanistic hypothesis. Autoimmunity, melanocyte adhesion, and metabolic dysfunction have been suggested as contributing to the etiology of vitiligo, and several genes based on the above mechanisms have been tested.^31^ Noteworthy examples include the DDR1 gene, which encodes a transmembrane tyrosine kinase receptor that is the main adhesion protein of melanocytes to the basement membrane and has been shown to be downregulated in vitiligo in comparison to unaffected skin.^32^, ^33^ DDR1 forms a complex with E-cadherin (encoded by *CDH1*), which is important for the maintenance of the epithelial structure. Variants close to the *CDH1* gene have been associated with vitiligo in Brazilian individuals, linking defects in melanocyte adhesion to the pathogenesis of vitiligo.^34^ Melanocyte damage due to excessive oxidative stress is another well-established candidate mechanism for vitiligo. The accumulation of reactive oxygen species in the epidermis can inhibit the BCHE enzymatic activity.^35^ Variants that control the enzymatic activity of the *BCHE* gene have been associated with vitiligo in independent samples of the Brazilian population.^36^

The main genetic changes linked to vitiligo are summarized in Table 1.

**Table 1:** Main genes and histocompatibility antigens (HLA) involved in the pathogenesis of vitiligo.

**Gene**	**Expression**
NLRP1	**+**
XBP1	**+**
FOXD3	**+**
*PDGFRA*	**+**
*PTPN22*	**+**
*IKZF4*	**+**
*FOXP3*	**+**
DDR1	-
**HLA**	**Risk**
HLA-A*02	**↑**
HLA-Aw*31	**↑**
HLA-A*32	**↑**
HLA-A*33	**↑**
HLA-A*09	**↓**
HLA-Aw*19	**↓**
HLA-DQB1*06	**↑**
HLA-DQB1*0303	**↑**
HLA-DR4	**↑**
HLA-DRB1*07	**↑**
HLA-DR7	**↑**

## Morphofunctional changes

In addition to the significant reduction in melanin and melanocytes, the skin with vitiligo shows morphological changes both in the epithelium and in the upper dermis, which supports the hypothesis that other elements contribute to disease development, in addition to melanocyte susceptibility to oxidative and immunological damage.

**Figure 1:**
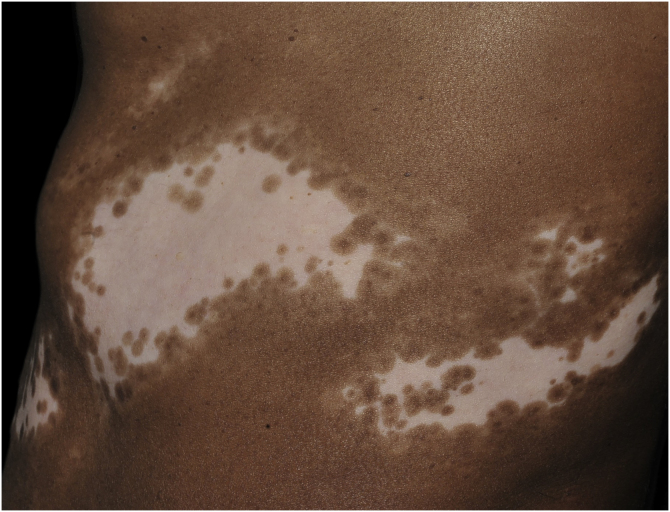
Segmental vitiligo on the flank of a dark-skinned patient. Achromic macula, with zosteriform distribution; the periphery of the lesion shows a leukomelanoderma band, with several follicular repigmentation points – Source: authors’ file.

**Figure 2:**
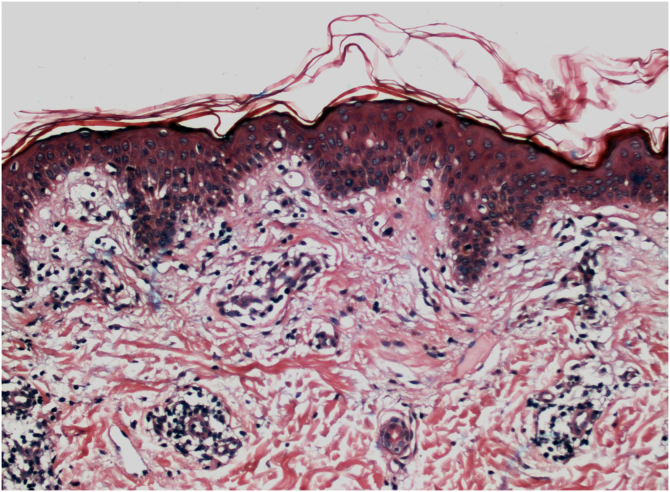
Active vitiligo. Histopathology: perivascular lymphocytic infiltrate, with epidermal aggression and basal layer vacuolar degeneration foci (Hematoxylin &eosin, ×40). Picture is a courtesy of Dr. Lismary Mosque.

Histopathologically, less pigmentation in the basal layer is observed in up to 78% of the epidermis with vitiligo, and some inflammatory infiltrate is identified in up to 48% of cases. In vitiligo cases with active disease, histopathology may show a lichenoid interface dermatitis pattern, demonstrating the focus of self-aggression located in the basal layer.^37^ T-lymphocytes (CD3+), especially the cytotoxic phenotypes (CD8+), are the predominant cells (65.4%) in the infiltrate, which is more evident in perilesional skin with perivascular and periannexal distribution. For this reason, perilesional skin is considered the area with the highest inflammatory activity in vitiligo (Figure 1, Figure 2).^38^

Screening of melanin pigmentation using Fontana-Masson staining identified residual melanin in 16% of vitiligo cases, and in 12% of cases, melanocytes were identified in the lesions, demonstrating that total destruction of melanocytes may not occur in the affected area.^38^

On electron microscopy, abnormal melanogenesis was identified in active vitiligo; melanocytes from the control skin and perilesional area of ​​stable vitiligo lesions showed long, thin dendrites with a moderate number of melanosomes, in contrast to the melanocyte dendrites from the perilesional area of ​​active vitiligo: retractable with few melanosomes.^39^ Langerhans cells are increased in the epidermis, the basement membrane is thickened, and degenerative alterations (e.g., cytoplasmic vacuolization) in basal melanocytes and keratinocytes are evidenced during disease activity.

The role of the remaining melanocytes in the basal layer in the repigmentation process of vitiligo is still unclear, especially because melanogenesis is compromised in the lesions. However, the maintenance of hair follicle pigmentation in an affected area indicates good prognosis since the migration of melanocytes from the outer follicular sheath can be evidenced by the identification of perifollicular repigmentation after phototherapy (Fig. 1).

The main source of repigmentation in vitiligo is the melanoblasts of the hair outer root sheath, when not attacked by CD8+ T-cells. After stimulation with phototherapy, these melanoblasts migrate, differentiate into melanocytes, and proliferate into the epidermis, probably through the upregulation of the stem cell-associated gene, *GLI1*.^40^

The stratum corneum and viable epidermis of vitiligo lesions are increased in thickness compared to non-lesional skin.^41^ The lesional epithelium also has larger corneocytes, likely to compensate for the decreased expression of cornification components and, therefore, they result in a thicker stratum corneum in vitiligo lesions. A possible explanation for this fact may be the reduced expression of D3 gangliosides, which favors keratinocyte apoptosis in patients with vitiligo. This potentially induces a compensatory mechanism of epidermal thickening to protect the affected skin against ultraviolet radiation (UVR).

However, previous optical and electron microscopy studies have demonstrated degenerative changes in keratinocytes in both affected and unaffected skin of patients with vitiligo,^42^ suggesting that the entire epithelium of patients with vitiligo is susceptible to and suffers from the pathogenic pressures of the disease.

In common vitiligo, there is a decrease in the epithelial cones of the dermal-epidermal junction. Similarly, a difference in architecture was identified in patients with segmental and nonsegmental types of vitiligo. In the segmental type, there is a notable increase in epithelial cones; acanthosis is observed in the nonsegmental type when compared with the skin of controls without vitiligo.^37^

Adhesion defects between cell components of the epidermis have also been implicated in the pathogenesis of vitiligo. E-cadherin is a protein that assists in anchoring between keratinocytes, and its low expression has been identified in melanocytes in vitiligo.^43^ It was also demonstrated in the dermis, p53-positive cells in non-lesional areas, and this reactivity was higher in vitiligo lesions than in controls.

The reduction of melanocytes in vitiligo was also related to a defect in cell adhesion but not directly to apoptosis. Furthermore, cytokines such as IFNγ and TNFα induce melanocyte detachment and decrease the distribution of E-cadherin in melanocytes. However, the combination of the two cytokines was able to downregulate the expression of the *CDH1* gene that encodes E-cadherin and also reduced the expression of the genes associated with adhesion deficit, *DDR1* and *CCN3*. Furthermore, MMP-9 is elevated in the skin and plasma of patients with common vitiligo, being produced in keratinocytes by the stimulation of TNFα and IFNγ and associated with E-cadherin cleavage.^44^

The DKK1 protein, which reduces melanogenesis and certain somatic functions of melanocytes, is overexpressed by lesional fibroblasts in vitiligo.^45^ Similarly, there is a greater expression of fibronectin, a key protein in intercellular communication by binding to integrins, in the dermis of patients with vitiligo compared with healthy controls.^43^ Additionally, elastin is reduced in lesional dermis, but collagen fibers show no difference when compared to unaffected skin.^46^

## Oxidative changes

Cutaneous melanocytes, located in the organ with the greatest interface with the external environment and, consequently, the most exposed to ultraviolet radiation and pollutants, are particularly vulnerable to excessive production of reactive oxygen species (ROS). The concentration of these substances is even higher than in other adjacent cells, such as keratinocytes and fibroblasts. This is due, in part, to their specialized function of producing melanin (which generates by-products such as O_2_^−^ and H_2_O_2_) and inflammation due to excessive photo exposure.^47^, ^48^

In the hair follicle, the outer sheath melanocytes are responsible for intense melanin synthesis. There is evidence that the production of ROS in these melanocytes favors white hairs during the aging process, which is also accompanied by loss of protective antioxidant mechanisms, generating an alteration in the oxidative/antioxidative balance (redox status). In an *in vitro* model of oxidative stress, glutamine (a precursor amino acid of the antioxidant molecule glutathione) was shown to be able to reduce melanocyte apoptosis. In addition to triggering apoptosis, ROS also has the potential to reduce melanogenesis, as demonstrated by *in vitro* tests following exposure of melanocytes to H_2_O_2_.^48^, ^49^, ^50^, ^51^

The theory of vitiligo development due to oxidative stress (OS) suggests that ROS production would be induced by multiple intrinsic factors (such as inflammation and protein synthesis), as well as extrinsic ones, such as exposure to ultraviolet radiation pollutants, and phenolic compounds. In parallel, a failure in the antioxidant mechanism would occur, disrupting cell homeostasis and culminating in cell damage.^52^

Melanocytes cultured from unaffected areas in patients with vitiligo have a greater susceptibility to oxidative stress than from controls without vitiligo, demonstrating an overall susceptibility of the patient with vitiligo to oxidative damage.^53^

There is an increase in the superoxide dismutase enzyme (responsible for degrading the O_2_^−^ radical into H_2_O_2_ and O_2_), an increase in lipid peroxidation (secondary to OS), in addition to a reduction in the catalase enzyme (which converts H_2_O_2_ into H_2_O and O_2_) in vitiligo. However, when compared to other inflammatory diseases such as psoriasis and lichen planus, such alterations are also reported, suggesting that the OS pathway is not disease-specific. In contrast, when non-lesional skin is assessed, there are more oxidative pathway alterations in the skin of patients with vitiligo than of patients with other inflammatory skin diseases, suggesting that the depigmented areas are a phenotypically altered part of skin subjected to oxi-reducing imbalance.^54^

In addition to having a lower systemic antioxidant capacity (glutathione peroxidase reduction) when compared to individuals without vitiligo,^55^ there are differences in the serum levels of superoxide dismutase (SOD) and reduced glutathione (GSH) between patients with common and localized vitiligo, also assuming a difference in the systemic antioxidant status according to disease severity.^56^ Polymorphisms of *FOXO3A*, a gene with an important role in OS regulation, are also found in patients with active vitiligo.^57^

Treatment with narrowband UVB, in turn, is able to balance the redox status in patients with vitiligo, as demonstrated by the significant reduction in serum malondialdehyde (MDA) levels and an increase in glutathione peroxidase in patients treated by this phototherapy method.^58^

It is hypothesized that OS inflicts cell damage by inducing apoptosis in melanocytes, as well as other cell death mechanisms, such as ferroptosis and phagoptosis.^52^ Clinical and biochemical studies suggest that the overexpression of the *TRPM2* gene (transient receptor potential cation channel subfamily M member 2) and the CGRP (calcitonin gene-related peptide) peptide are related to OS-sensitive calcium channels in perilesional melanocytes in vitiligo. In these cases, H_2_O_2_ would induce the demethylation of the promoter region of the *TRPM2* gene and increase its expression in melanocytes, promoting the calcium influx into the cytoplasm, and promoting its apoptosis.^59^

In a study with murine melanocytes, the natural process of autophagy, in which cells degrade their organelles and damaged proteins to maintain their homeostasis, was also shown to be impaired when excessive ROS accumulation occurs.^60^ In vitiligo, melanocytes and fibroblasts are more sensitive to OS-induced autophagy, which reduces their potential to control melanogenesis.^61^

OS can also trigger changes in the melanocyte endoplasmic reticulum, culminating in the accumulation of defective proteins, stimulating a cell stress phenomenon called “unfolded protein response” (UPR).^62^ Moreover, energy requirements for protein production (such as melanin) alone generate ROS through mitochondrial metabolism. These two pathways seem to be overactivated in the melanocytes of patients with vitiligo, suggesting that these cells tolerate less the demand for melanin production than those of healthy individuals.^63^ Even healthy melanocytes undergo cell stress when exposed to certain phenolic compounds such as hydroquinone monobenzyl ether, inducing the production of IL6 and IL8.^63^, ^64^

Despite the extensive use of antioxidants by patients with vitiligo who seek therapeutic alternatives, the role of this pathogenic mechanism and the application of this knowledge in clinical practice remain controversial. The lack of a consistent response to systemic antioxidants suggests that OS may not play a central role in disease pathogenesis, which raises the need for studies to identify the real role of the mechanism in this complex disease.^54^

**Figure 3:**
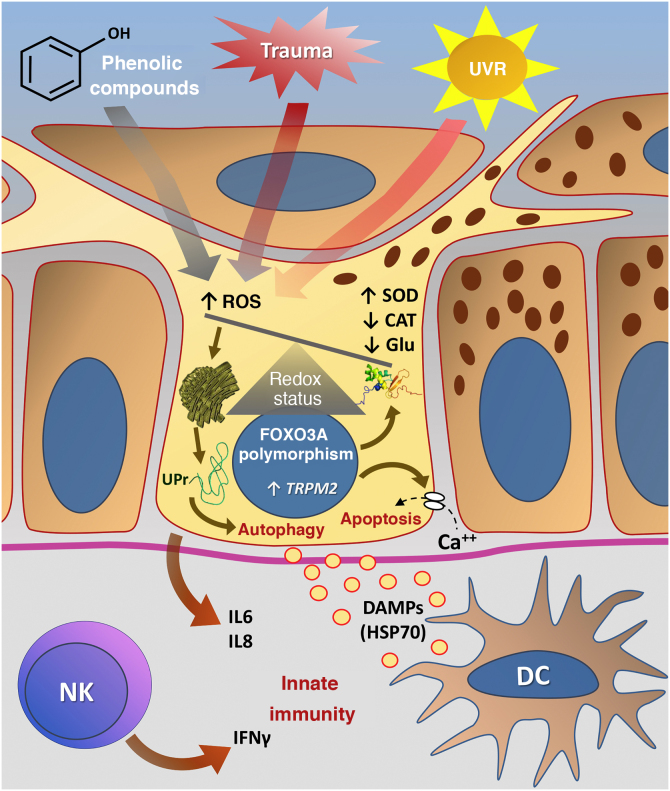
Representation of Oxidative Stress (OS) and activation of innate immunity in vitiligo. The effects of ultraviolet radiation (UVR), phenolic compounds and trauma (Köbner) increase the production of reactive oxygen species (ROS). In parallel, genetic predisposition (such as mutations in the *FOXO3A* gene) lead to the inefficiency of antioxidant mechanisms, observed by an increase in the superoxide dismutase (SOD) enzyme, reduction in catalase (CAT) and glutathione (Glu), causing an imbalance in the redox status. OS also causes an accumulation of defective proteins in the endoplasmic reticulum, resulting in a phenomenon called the response to unfolded proteins (UPr), contributing to the process of autophagy leading to the production of proinflammatory interleukins (IL6 and IL8). The increased expression of TRPM2 (transient receptor potential cation channel subfamily M member 2), also induced by OS, promotes an influx of calcium into the melanocyte, culminating in its apoptosis. The OS promotes the release of DAMPs (damage-associated molecular patterns), especially HSP70, which initiate the innate response from the activation of dendritic cells (DC) and the participation of NK cells (natural killer) – Source: the authors.

Fig. 3 summarizes the possible pathways of damage to melanocytes due to OS in vitiligo.

In vitiligo, OS also participates in the Köbner phenomenon, although its etiology is probably multifactorial. After epithelial trauma, inflammatory mediators lead to an increase in OS, inducing cell death, a process that is also favored by melanocyte adhesion defects. Additionally, epithelial trauma promotes the release of IFNα, which increases the expression of CXCL10, favoring the migration of circulating lymphocytes.^65^

## Immunological changes

### Innate immunity

Evidence for the participation of the innate immune system in the pathogenesis of vitiligo is very consistent, with the latter being considered the link between oxidative stress and the adaptive immune response.^66^ First, dendritic cells, macrophages, and NK (natural killer) cells are found in the lesional and perilesional skin of patients with vitiligo, characterizing an activation of the innate immune response.^67^

Activated NK cells (CD56+ / granzyme B+) and IFNγ-producing cells have been identified in the blood and non-lesional skin of patients with vitiligo.^68^ Moreover, there is an increase in proinflammatory cytokines, characteristic of innate immunity, in both serum and skin of patients with vitiligo, such as IL1α, IL1β, IL6, IL8, IL12, IL15, and TNFα.^69^ These elements indicate a global activation of the body innate immunity, transcending the affected areas.

The oxidative stress that occurs in the melanocyte, as mentioned before, is possibly the autoimmunity trigger in vitiligo.^63^ The communication between the melanocyte and the innate immune system seems to occur through the secretion of exosomes, which contain melanocyte-specific antigens, miRNAs, heat shock proteins, and DAMPs (damage-associated molecular patterns).^64^ These exosomes deliver autoantigens to dendritic cells, which undergo maturation into antigen-presenting cells.^70^

The DAMP with the highest evidence of association with vitiligo to date is HSP70 (heat shock protein 70).^66^ HSP70 is a protein from the intracellular chaperone family whose function is to prevent the incorrect folding of other proteins and their aggregation. Whereas some HSPs facilitate the folding of newly formed proteins, others are particularly induced during physiological stress to manage the extra burden of stress-induced protein misfolding, thus preserving cell viability.^71^ Although HSPs are intracellular proteins, it has been observed that, under stress situations, they can be secreted by the cell. In the extracellular environment, the identification of these proteins by cells of the immune system results in signal transduction that leads to the release of proinflammatory cytokines, such as IFNα. *In vitro* studies have demonstrated the expression of HSP70 Lox-1 receptors on the surface of dendritic cells in vitiligo. The same study showed that HSP70 induces the maturation and activation of dendritic cells (CD80+).^71^, ^72^

The induced HSP70 is present in lesional and perilesional skin in vitiligo. Studies in animal models have demonstrated the relationship between induced HSP70 and the recruitment and activation of inflammatory dendritic cells in vitiligo. Furthermore, in rats, HSP70 has been shown not only to be necessary but sufficient to accelerate skin depigmentation.^73^ Finally, a study that introduced a mutant HSP70, in which only one amino acid was altered in its structure, showed that it was able to promote skin repigmentation by binding to dendritic cells and inactivating them and, consequently, inhibiting the subsequent T-cell response. This HSP70 molecule with an amino acid change is considered a potential treatment for vitiligo and is expected to be studied in clinical trials.^74^

NLRP-1 (nuclear localization leucine-rich-repeat protein 1) is another component of innate immunity identified in vitiligo. Variants of the DNA sequence in the NALP1 region are associated with the risk for several epidemiologically associated autoimmune and autoinflammatory diseases, including common vitiligo.^9^ NLRP-1 is a key regulator of the innate immune response. By recognizing the DAMPs, this receptor activates the inflammasome, which, through the caspase-1 pathway, induces the processing of pro-IL1β into active IL1β, as well as its secretion into the extracellular environment, where it perpetuates the inflammatory response.^75^ IL1β plays a key role in the polarization of T cells towards Th17.^76^ The perilesional skin of vitiligo, where the disease is most active, shows an increase in IL1β, suggesting that this pathway is also involved in vitiligo progression.^77^ Therefore, IL1β inhibition can also be seen as a potential therapeutic target in vitiligo.

### Adaptive immunity

The activation of innate immunity triggered by damage to melanocytes affected by oxidative stress promotes cytokine secretion and antigen presentation, resulting in the adaptive immune system activation, in which autoreactive T-cells amplify damage to melanocytes in vitiligo-affected skin.

In this context, cytotoxic T-lymphocytes (CD8+) are the main cells implicated in disease pathogenesis.^78^ However, although antibodies reactive against melanocytes (e.g., anti-MelanA, anti-MCHR1, anti-tyrosinase, anti-gp100, and anti-tyrosine hydroxylase) have elevated serum titers in patients with vitiligo, they do not correlate with disease activity.^79^ Nevertheless, the literature is controversial, and the exact role of anti-melanocyte antibodies in vitiligo remains unclear.

The type I IFN pathway is a key point for the start of the vitiligo immune response. The IFN-I signature characterizes an early and transient event in its progression and a link between the innate and adaptive immune response.^65^ Studies have shown that IFNα, produced mainly by plasmacytoid dendritic cells (pDCs), stimulates the production of chemokines, such as CXCL9 and CXCL10, by keratinocytes, leading to the recruitment of T-cells expressing their receptor: CXCR3.^65^, ^72^ Furthermore, HSP70 potentially contributes to skin inflammation by activating pDCs and increasing IFNα secretion.^72^

Cytotoxic CD8+ T-cells are necessary and sufficient for the destruction of melanocytes, acting as an effector arm of autoimmunity.^78^ The lesions are caused by effector CD8+ T-lymphocytes in the initial or active phase of the disease and by recirculating and resident memory CD8 + T (TEM) lymphocytes in the stable phase. The mechanism of aggression is based on the production of inflammatory cytokines such as TNFα and IFNγ, in addition to the release of granzymes and perforins, which cause direct damage to melanocytes.^78^, ^80^ In the lymphocytic infiltrate of the periphery of the depigmented areas, CD8+ T-lymphocytes predominate, and this finding correlates with disease activity.^81^

Melanocyte-specific antigens recognized by CD8+ T-cells, such as MelanA, tyrosinase, gp100, and tyrosinase-related proteins 1 and 2, are detected in greater numbers in the peripheral blood of patients with vitiligo when compared to controls.^82^ The perilesional skin also expresses higher levels of CD8+ T lymphocytes against melanocyte antigens in comparison to normal skin.^78^ These autoreactive cells are able to destroy melanocytes *in vitro*^81^ in addition to inducing apoptosis of keratinocytes and melanocytes in a pattern similar to the disease.^78^

IFNγ, and the genes it induces, encode the CXCR3 chemokine receptor and its ligands CXCL9 and CXCL10, which are critical for the activation of CD8+ T cells, and are found to be increased in the skin and blood of patients with vitiligo.^83^, ^84^ CXCL9 promotes the global recruitment of autoreactive T-cells but with no effector action, whereas CXCL10 is required for disease progression and maintenance.^85^ Moreover, the neutralization of CXCL10 in animal models prevents the appearance of new lesions and induces the repigmentation of established areas,^85^ identifying the therapeutic potential of IFNγ/CXCL10/CXCR3 axis inhibition.^86^ Keratinocytes are the main sources of these cytokines, and CXCL9 and CXCL10 measurements are potential biomarkers of disease activity.^84^

The Janus kinase/signal transducers and transcription activators (JAK/STAT) pathway participate in the immunopathogenesis of vitiligo through its interaction with IFNγ. IFNγ/CXCL10 signaling starts with the binding of IFNγ to its heterodimeric receptor, which stimulates the JAK/STAT pathway and leads to STAT1 activation. Subsequently, STAT1 translocation to the nucleus and subsequent binding to the IFNγ-induced gene promoter region such as CXCL10 occur. There are four members of the JAK protein family, including JAK1, JAK2, JAK3, and tyrosine kinase 2 (TYK2). JAK1 and JAK2 are directly involved in IFNγ signaling. In this context, some studies have used several JAK inhibitors as a repigmentation strategy.^87^

Vitiligo recurrence is a common phenomenon, often occurring in the same affected site prior to treatment. This pattern of recurrence suggests the role of autoimmune memory in the maintenance of the condition, characterized by the presence of memory CD8 + T Cells in both the epidermis and dermis, which act as sentinels for the recruitment of T-effector memory cells from the circulation.^80^ TRMs are long-lived lymphocytes that develop after the start of a T-cell-mediated immune response.

Based on the demonstration that TRMs require IL15 for their differentiation, the interruption of the IL15 pathway using an anti-CD122 antibody caused reversal of depigmentation in mice with stable vitiligo. In that trial, short-term treatment with anti-CD122 inhibited IFN-γ production by TRMs, and long-term use depleted these cells from the lesions.^88^ Additional IL15 functions have also been mapped in other studies. One study showed that IL15 causes the expression of NKG_2_D in memory CD8 + T Cells in skin with vitiligo, triggering the production of IFNγ and TNFα.^89^ Additionally, oxidative stress has been shown to induce IL15 transpresentation in keratinocytes, contributing to the activation of memory CD8 + T Cells through the activation of the JAK/STAT pathway (JAK1-STAT3 and JAK3-STAT5).^90^ Thus, blocking IL15 signaling seems to be promising in the search for new treatments.

**Figure 4:**
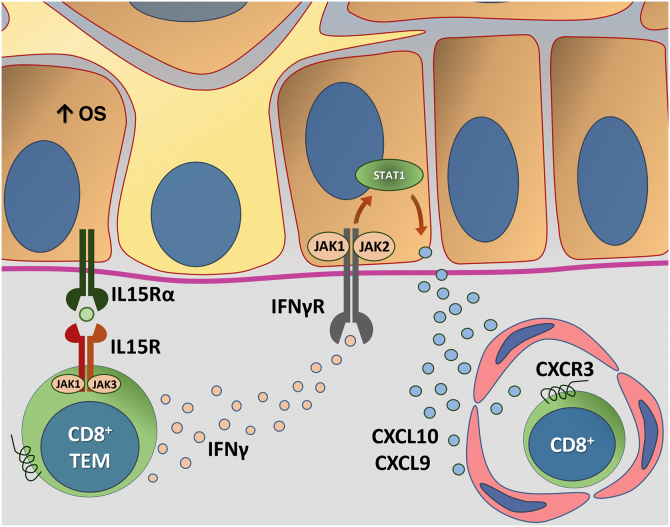
Representation of Interleukin (IL)-15 transpresentation in keratinocytes induced by oxidative stress (OS) and the interaction of interferon gamma (IFNγ) with the Janus kinase/signal transducers and transcription activators (JAK/STAT) pathway. The OS promotes the transpresentation of I-15 in keratinocytes through the binding of IL-15 to the heterodimeric IL-15 receptor (IL15R) on memory CD8 + T lymphocytes, consisting of CD122 and CD132, and to the I-15α receptor (IL15Rα) on keratinocytes (CD215). This process potentiates the activation of memory CD8 + T Cells and the production of inflammatory cytokines, such as IFNy, via JAK/STAT signaling (JAK1-STAT3 and JAK3-STAT5). The IFNγ/STAT1/CXCL10 axis conducts the autoimmune destruction of melanocytes. IFNγ signals through the IFNγ receptor (IFNγR) to stimulate JAK1/JAK2 and activate STAT1. The activation induces the production of CXCL9 and CXCL10, which signals through the CXCR3 receptor for the recruitment of more autoreactive CD8+ T cells – Source: the authors.

Fig. 4 details the IL15 transpresentation in keratinocytes induced by oxidative stress and the interaction of IFNγ with the JAK/STAT pathway.

CD4+ T-regulatory cells (Tregs) act to maintain tolerance to their own tissues by suppressing the activity of T-effector cells. In vitiligo, there is a Treg dysfunction, although it is not known exactly whether due to the inability to migrate to the skin, decreased numbers, or activity suppression.^91^ In animal models of vitiligo, there is control of disease progression and repigmentation of lesions with the re-establishment of the Treg population.^92^

Alterations of the adaptive immune system are demonstrated in both segmental and nonsegmental vitiligo, despite different reactivity patterns. In nonsegmental vitiligo, systemic immune activation occurs, whereas in the segmental type, there is only a localized cytotoxic reaction, probably due to melanocyte mosaicism.^93^ Tregs are unaffected in the peripheral blood of segmental vitiligo when compared to controls. On the other hand, Tregs are reduced in nonsegmental vitiligo and associated with other autoimmune comorbidities, less frequent in segmental vitiligo. Moreover, the antibody response against melanocyte-specific antigens occurs only in nonsegmental vitiligo.

The higher frequency of autoimmune comorbidities in patients with nonsegmental vitiligo and also in their relatives reinforces the idea of ​​vitiligo as a phenotypic representation of a systemic autoimmune imbalance. Comorbidities vary widely with the studied population and depend on sex, age, ethnicity, and vitiligo subtype. Table 2 lists the main autoimmune and autoinflammatory diseases described in vitiligo, with autoimmune thyroidites being the most frequent and well-established ones. Also noteworthy are ocular and auditory abnormalities due to the presence of melanocytes in the uvea and cochlea.^3^, ^94^

**Table 2:** Main autoimmune and auto-inflammatory diseases associated with vitiligo.

**Autoimmune or auto-inflammatory disease**
Alopecia areata
Pernicious anemia
Rheumatoid arthritis
Atopic dermatitis
Dermatomyositis
Type 1 Diabetes Mellitus
Addison’s disease
Crohn’s disease
Graves’ disease
Systemic scleroderma
Multiple sclerosis
Systemic lupus erythematosus
Psoriasis
Ulcerative colitis
Sjogren’s Syndrome
Hashimoto’s Thyroiditis

The development of vitiligo and halo nevus in patients with melanoma is a well-described phenomenon, probably due to auto aggression to melanocytes induced by tumor antigens. However, this occurs in 10% to 25% of patients treated with checkpoint inhibitors (e.g., nivolumab, pembrolizumab), a new class of medication for metastatic melanoma. These clinical lesions are indistinguishable from the common vitiligo, and the phenomenon is called leukoderma or vitiligo-like lesions; however, there is some doubt as to whether their origin is due to direct cytotoxicity to melanocytes or the development of autoimmunity (vitiligo) in predisposed patients.^95^

**Figure 5:**
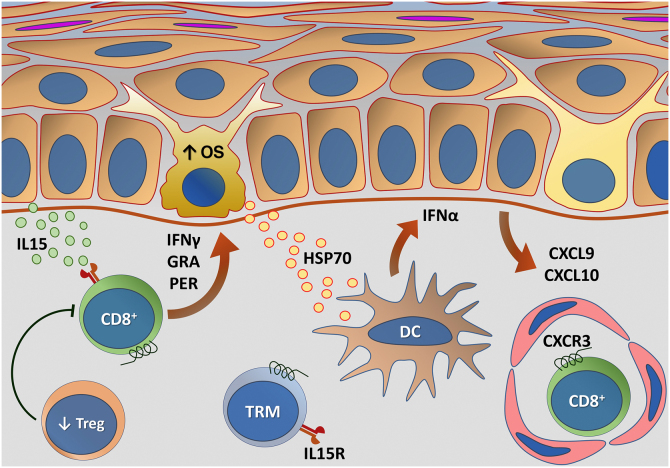
Representation of alterations related to adaptive immunity in vitiligo. Melanocytes affected by oxidative stress (OS) trigger the activation of innate immunity through the secretion of exosomes, which contain damage-associated molecular patterns (DAMPs), especially heat shock protein 70 (HSP70). HSP70 stimulates the secretion of IFNα by dendritic cells in the initial phase of disease progression, which induces the production of chemokines CXCL9 and CXCL10 by keratinocytes and the recruitment of T-cells expressing the CXCR3 receptor. CXCL10 has an effector action, while CXCL9 acts on the global recruitment of autoreactive CD8+ T-cells. Effector CD8+ T-cells are responsible for the destruction of melanocytes through the production of interferon gamma (IFNγ), release of granzymes and perforins, facilitated by T- regulatory (Treg) cell dysfunction. CD8+ tissue-resident memory T cells (TRM) develop after the onset of the T-cell-mediated immune response and are implicated in disease maintenance, being retained in the tissue due to IL15 transpresentation by keratinocytes – Source: the authors.

Fig. 5 outlines the main changes in adaptive immunity involved in vitiligo.

The exact mechanism by which topical therapies with corticosteroids and calcineurin inhibitors affect the disease pathogenesis has yet to be fully elucidated, but it is believed that they reduce the lymphocytic infiltrate and TNFα expression. In trauma-induced injuries (Köbner), treatment with topical corticosteroids and tacrolimus reduced the underlying inflammatory infiltrate.^96^

Phototherapy, in addition to stabilizing the redox balance, suppresses the inflammatory response, promoting T-cell apoptosis, decreasing inflammatory cytokines, increasing IL10 (with regulatory T-cell induction), reducing JAK1 expression, and depleting the Langerhans cells of the epidermis.^97^

## Vitiligo induced by chemical agents

Chemical-associated vitiligo, also called leukoderma, has been described especially in rubber industry workers in contact with phenolic compounds. The first identified agent was hydroquinone monobenzyl ether (monobenzone), which induces cell death without activating the caspase cascade or DNA fragmentation.^98^

Rhododendrol, another phenolic compound used in skin lightening treatments responsible for an outbreak of leukoderma in Japan, promotes cytotoxicity via a tyrosinase-dependent mechanism.^99^

Hair dyes have also been implicated in the appearance of leukoderma, and one of the main suspects is paraphenylenediamine. However, the causal mechanism by which benzene derivatives cause depigmentation has yet to be elucidated.^100^

Chemical leukoderma, however, does not share the autoimmunity changes (e.g., anti-TYRP1 antibody) as it occurs in vitiligo, despite promoting direct damage to melanocytes.^101^

## Neural theory

The neural theory of vitiligo is based on the clinical observation of lesion distribution: on dermatomes in the segmental type and symmetrical in nonsegmental vitiligo, alluding to the neuroimmunological influence in vitiligo. There are also anecdotal cases of vitiligo appearing in delimited areas after nerve injury.^102^ Finally, extreme psychological stress is a factor classically associated with the development of vitiligo and other autoimmune diseases; although the pathophysiological mechanism involved in this context is not fully elucidated, whether dependent on the change in the oxidative state, the modification of the immune tolerance system, or the participation of neurokinins.^103^

In vitiligo, neuropeptide Y is increased in lesional skin and the perilesional area.^104^ Substance P is increased in the skin of stable vitiligo when compared to unstable disease, suggesting that it is associated with vitiligo stability.^105^

Extreme physical or psychological stress increases the synthesis of catecholamines and requires the activation of the hypothalamic-pituitary-adrenal axis, which interferes with the immune system regulation. In the acute stress phase, neutrophilia occurs, and there is an increase in NK cells in the plasma, in addition to proinflammatory cytokine imbalance, with an increase in IL6, resulting from the secretion of cortisol and catecholamines, modulating the immune response and the development of autoimmune and autoinflammatory diseases.^106^

## Emerging therapies

The three emerging vitiligo therapeutic classes that are at the most advanced stage of development and are associated with the most important pathogenic pathways are the tyrosine kinase inhibitors (e.g., JAK1, JAK2, JAK3, TYK2), anti-IL15, and mutant HSP70.^74^, ^88^, ^107^ Table 3 shows the emerging drugs according to their pathophysiological target.

**Table 3:** Main emerging therapies and therapeutic targets in vitiligo.

Class / Drug	Therapeutic target/ Action mechanism
Mutant HSP70 protein	Innate immunity inhibition ‒ reduced dendritic cell activation
HSP70iQ435A	
	
Tyrosine kinase inhibitors	Adaptive immunity ‒ alteration in cytokine/chemokine signaling (IFNy, CXCL10, IL15)
Tofacitinibe (JAK1/3)	
Ruxolitinibe (JAK1/2)	
Cerdulatinib (SYK/JAK 1/2/3)	
ATI-50002 (JAK1/3)	
PF-06651600 (JAK3)	
PF-06700841 (TYK2/JAK1)	
	
Anti-IL15 Biologicals	Adaptive immunity – blocking of IL15 signaling
Anti-CD122 mAb	
AMG 714 (Anti-IL15 mAb)	

HSP70, heat shock protein 70; JAK, Janus kinase; SYK, spleen tyrosine kinase; TYK, tyrosine kinase; IFNy, interferon gama; IL, interleukin; mAb, monoclonal antibody.

## Conclusion and future research

The understanding of the pathogenesis of vitiligo develops in parallel with the knowledge of the genetic regulation of the phenomena linked to OS and the cutaneous immune response.

The epigenetic control of the gene expression linked to these phenomena can be an integrator of these processes since environmental and dietary factors, behavioral response patterns to stress, and the increasing exposure to industrialized compounds are intensely present in modern life, especially air pollution and aromatic compounds.^108^, ^109^

The regulation of pathways related to transport vesicles (exosomes) between keratinocytes, melanocytes, and inflammatory cells constitutes another factor that should elucidate the interaction between the cell damage process, melanogenesis, and the immune response in vitiligo.^110^

The role of intestinal dysbiosis in inflammation modulation, OS, and autoimmunity may disclose susceptibility profiles and the clinical expression of vitiligo, as increased intestinal permeability favors the activation of innate immunity and promotes systemic inflammatory stimulation.^111^

Finally, the importance of hypovitaminosis D, a modern epidemic, in the immune response regulation, as well as the effect of its supplementation as an adjunct to treatment, must be explored since patients with vitiligo have lower levels of vitamin D.^112^

In conclusion, the pathogenesis of vitiligo results from an interaction of elements (multifactorial), which suggests a genetic susceptibility basis, affected by environmental factors, oxidative stress, psychological factors, and patterns of autoimmune response. The integration of these theories is the main challenge in the construction of pathophysiological models.

## Financial support

None declared.

## Authors’ contributions

Helena Zenedin Marchioro: Approval of the final version of the manuscript; drafting and editing of the manuscript; critical review of the literature; critical review of the manuscript.

Caio César Silva de Castro: Approval of the final version of the manuscript; design and planning of the study; drafting and editing of the manuscript; critical review of the literature; critical review of the manuscript.

Vinicius Medeiros Fava: Approval of the final version of the manuscript; drafting and editing of the manuscript; critical review of the literature; critical review of the manuscript.

Paula Hitomi Sakiyama: Approval of the final version of the manuscript; drafting and editing of the manuscript; critical review of the literature; critical review of the manuscript.

Gerson Dellatorre: Approval of the final version of the manuscript; drafting and editing of the manuscript; critical review of the literature; critical review of the manuscript.

Hélio Amante Miot: Approval of the final version of the manuscript; design and planning of the study; drafting and editing of the manuscript; critical review of the literature; critical review of the manuscript.

## Conflicts of interest

Dr. Caio Castro: Advisory Board – Abbvie, Sun Pharma and Aché. Dr. Hélio Miot: Advisory Board – L’Oréal; Merz.

## References

[1] Zhang Y., Cai Y., Shi M., Jiang S., Cui S., Wu Y. (2016). The prevalence of vitiligo: a meta-analysis. PLoS One.

[2] Castro CCS, Miot HA (2018). Prevalence of vitiligo in Brazil—a population survey. Pigment Cell Melanoma Res.

[3] Castro C.C.S., Nascimento L.L.M., Olandoski M., Mira M.T. (2012). A pattern of association between clinical form of vitiligo and disease-related variables in a Brazilian population. J Dermatol Sci.

[4] Boza J.C., Giongo N., Machado P., Horn R., Fabbrin A., Cestari T. (2016). Quality of life impairment in children and adults with vitiligo: a cross-sectional study based on dermatology-specific and disease-specific quality of life instruments. Dermatology.

[5] Dellatorre G., Antelo D.A.P., Bedrikow R.B., Cestari T.F., Follador I., Ramos D.G. (2020). Consensus on the treatment of vitiligo — Brazilian Society of Dermatology. An Bras Dermatol.

[6] Arcos-Burgos M., Parodi E., Salgar M., Bedoya E., Builes J., Jaramillo D. (2002). Vitiligo: complex segregation and linkage disequilibrium analyses with respect to microsatellite loci spanning the HLA. Hum Genet.

[7] Zhang X.J., Liu J.B., Gui J.P., Li M., Xiong Q.G., Wu H.B. (2004). Characteristics of genetic epidemiology and genetic models for vitiligo. J Am Acad Dermatol.

[8] Spritz R.A., Gowan K., Bennett D.C., Fain P.R. (2004). Novel vitiligo susceptibility loci on chromosomes 7 (AIS2) and 8 (AIS3), confirmation of SLEV1 on chromosome 17, and their roles in an autoimmune diathesis. Am J Hum Genet.

[9] Jin Y., Mailloux C.M., Gowan K., Riccardi S.L., LaBerge G., Bennett D.C. (2007). NALP1 in vitiligo-associated multiple autoimmune disease. N Engl J Med.

[10] Jin Y., Birlea S.A., Fain P.R., Spritz R.A. (2007). Genetic variations in NALP1 are associated with generalized vitiligo in a Romanian population. J Invest Dermatol.

[11] Liang Y., Yang S., Zhou Y., Gui J., Ren Y., Chen J. (2007). Evidence for two susceptibility loci on chromosomes 22q12 and 6p21-p22 in Chinese generalized vitiligo families. J Invest Dermatol.

[12] Birlea S.A., Jin Y., Bennett D.C., Herbstman D.M., Wallace M.R., McCormack W.T. (2011). Comprehensive association analysis of candidate genes for generalized vitiligo supports XBP1, FOXP3, and TSLP. J Invest Dermatol.

[13] Singh A., Sharma P., Kar H.K., Sharma V.K., Tembhre M.K., Gupta S. (2012). HLA alleles and amino-acid signatures of the peptide-binding pockets of HLA molecules in vitiligo. J Invest Dermatol.

[14] Liu J.B., Li M., Chen H., Zhong S.Q., Yang S., Du WD (2007). Association of vitiligo with HLA-A2: a meta-analysis. J Eur Acad Dermatol Venereol.

[15] Yang C., Wu J., Zhang X., Wen L., Sun J., Cheng Y. (2018). Fine-mapping analysis of the MHC region for vitiligo based on a new Han-MHC reference panel. Gene.

[16] Hayashi M., Jin Y., Yorgov D., Santorico S.A., Hagman J., Ferrara T.M. (2016). Autoimmune vitiligo is associated with gain-of-function by a transcriptional regulator that elevates expression of HLA-A*02:01 in vivo. Proc Natl Acad Sci U S A.

[17] Chen J.J., Huang W., Gui J.P., Yang S., Zhou F.S., Xiong Q.G. (2005). A novel linkage to generalized vitiligo on 4q13-q21 identified in a genome-wide linkage analysis of Chinese families. Am J Hum Genet.

[18] Alkhateeb A., Stetler G.L., Old W., Talbert J., Uhlhorn, Taylor M (2002). Mapping of an autoimmunity susceptibility locus (AIS1) to chromosome 1p31.3-p32.2. Hum Mol Genet.

[19] Fain P.R., Gowan K., LaBerge G.S., Alkhteeb A., Stetler G.L., Talbert J. (2003). A genome-wide screen for generalized vitiligo: confirmation of AIS1 on chromosome 1p31 and evidence for additional susceptibility loci. Am J Hum Genet.

[20] Ramire L.D., Marcos E.V., Godoy D.A., de Souza-Santana F.C. (2016). Association of class I and II HLA alleles and haplotypes with susceptibility to vitiligo: a study of patients with vitiligo from southeast Brazil. Int J Dermatol.

[21] Li Z., Ren J., Niu X., Xu Q., Wang X., Liu Y. (2016). Meta-analysis of the association between vitiligo and human leukocyte antigen-A. Biomed Res Int.

[22] Jin Y., Andersen G., Yorgov D., Ferrara T.M., Ben S., Brownson K.M. (2016). Genome-wide association studies of autoimmune vitiligo identify 23 new risk loci and highlight key pathways and regulatory variants. Nat Genet.

[23] Quan C., Ren Y.Q., Xiang L.H., Sun L.D., Xu A.E., Gao H. (2010). Genome-wide association study for vitiligo identifies susceptibility loci at 6q27 and the MHC. Nat Genet.

[24] Jin Y., Birlea S.A., Fain P.R., Ferrara T.M., Bem S., Riccardi S.L. (2012). Genome-wide association analyses identify 13 new susceptibility loci for generalized vitiligo. Nat Genet.

[25] Tang X.F., Zhang Z., Hu D.Y., Xu A.E., Zhou H.S., Sun L.D. (2013). Association analyses identify three susceptibility Loci for vitiligo in the Chinese Han population. J Invest Dermatol.

[26] Jin Y., Birlea S.A., Fain P.R., Gowan K., Riccardi S.L., Holland P.J. (2010). Variant of TYR and autoimmunity susceptibility loci in generalized vitiligo. N Engl J Med.

[27] Chung SA, Criswell LA (2007). PTPN22: its role in SLE and autoimmunity. Autoimmunity.

[28] Rivas M.A., Beaudoin M., Gardet A., Stevens C., Sharma Y., Zhang C.K. (2011). Deep resequencing of GWAS loci identifies independent rare variants associated with inflammatory bowel disease. Nat Genet.

[29] Pan F., Yu H., Dang E.V., Barbi J., Pan X., Grosso J.F. (2009). Eos mediates Foxp3-dependent gene silencing in CD4+ regulatory T cells. Science.

[30] Roberts G.H.L., Paul S., Yorgov D., Santorico S.A., Spritz R.A. (2019). Family clustering of autoimmune vitiligo results principally from polygenic inheritance of common risk alleles. Am J Hum Genet.

[31] Tarlé R.G., Nascimento L.M., Mira M.T., Castro CCS (2014). Vitiligo—part 1. An Bras Dermatol.

[32] Ricard A.S., Pain C., Daubos A., Ezzedine K., Lamrissi-Garcia I., Bibeyran A. (2012). Study of CCN3 (NOV) and DDR1 in normal melanocytes and vitiligo skin. Exp Dermatol.

[33] Reichert-Faria A., Jung J.E., Moreschi Neto V., Castro C.C.S., Mira M.T., Noronha L. (2013). Reduced immunohistochemical expression of Discoidin Domain Receptor 1 (DDR1) in vitiligo skin. J Eur Acad Dermatol Venereol.

[34] Tarlé R.G., Castro C.C.S., do Nascimento L.M., Mira M.T. (2015). Polymorphism of the E-cadherin gene CDH1 is associated with susceptibility to vitiligo. Exp Dermatol.

[35] Schallreuter K.U., Gibbons N.C.J., Zothner C., Elwary S.M., Rokos H., Wood J.M. (2006). Butyrylcholinesterase is present in the human epidermis and is regulated by H2O2: more evidence for oxidative stress in vitiligo. Biochem Biophys Res Commun.

[36] Nascimento L.M., Castro C.C.S., Fava V.M., Werneck R.I., Mira M.T. (2015). Genetic and biochemical evidence implicates the butyrylcholinesterase gene BCHE in vitiligo pathogenesis. Exp Dermatol.

[37] Montes L.F., Abulafia J., Wilborn W.H., Hyde B.M., Montes C.M. (2003). Value of histopathology in vitiligo. Int J Dermatol.

[38] Kim Y.C., Kim Y.J., Kang H.Y., Sohn S., Lee E.S. (2008). Histopathologic features in vitiligo. Am J Dermatopathol.

[39] Xiong X.X., Ding G.Z., Zhao W.E., Li X., Ling Y.T., Sun Li (2017). Differences in the melanosome distribution within the epidermal melanin units and its association with the impairing background of leukoderma in vitiligo and halo nevi: a retrospective study. Arch Dermatol Res.

[40] Goldstein N.B., Koster M.I., Jones K.L., Gao B., Hoaglin L.G., Robinson S.E. (2018). Repigmentation of human vitiligo skin by NBUVB is controlled by transcription of GLI1 and activation of the beta-catenin pathway in the hair follicle bulge stem cells. J Invest Dermatol.

[41] Gniadecka M., Wulf H.C., Mortensen N.N., Poulsen T. (1996). Photoprotection in vitiligo and normal skin. A quantitative assessment of the role of stratum corneum, viable epidermis and pigmentation. Acta Derm Venereol.

[42] Bhawan J, Bhutani LK (1983). Keratinocyte damage in vitiligo. J Cutan Pathol.

[43] Kovacs D., Bastonini E., Ottaviani M., Cota C., Migliano E., Dell’Anna M.L. (2018). Vitiligo skin: exploring the dermal compartment. J Invest Dermatol.

[44] Boukhedouni N., Martins C., Darrigade A.S., Drullion C., Rambert J., Barrault C. (2020). Type-1 cytokines regulate MMP-9 production and E-cadherin disruption to promote melanocyte loss in vitiligo. JCI Insight.

[45] Rani S., Chauhan R., Parsad D., Kumar R. (2018). Effect of Dickkopf1 on the senescence of melanocytes: in vitro study. Arch Dermatol Res.

[46] Hirobe T., Enami H., Nakayama A. (2020). Elastin fiber but not collagen fiber is decreased dramatically in the dermis of vitiligo patients. Int J Dermatol.

[47] Denat L., Kadekaro A.L., Marrot L., Leachman S.A., Abdel-Malek Z.A. (2014). Melanocytes as instigators and victims of oxidative stress. J Invest Dermatol.

[48] Jenkins N.C., Grossman D. (2013). Role of melanin in melanocyte dysregulation of reactive oxygen species. Biomed Res Int.

[49] Jiang L., Guo Z., Kong Y., Liang J., Wang Y., Wang K. (2018). Protective effects of glutamine on human melanocyte oxidative stress model. Indian J Dermatol Venereol Leprol.

[50] Arck P.C., Overall R., Spatz K., Liezman C., Handjiski B., Kalpp B.E. (2006). Towards a “free radical theory of graying”: melanocyte apoptosis in the aging human hair follicle is an indicator of oxidative stress induced tissue damage. FASEB J.

[51] Jimenez-Cervantes C., Martinez-Esparza M., Perez C., Daum N., Solano F., Garcia-Borron J.C. (2001). Inhibition of melanogenesis in response to oxidative stress: transient downregulation of melanocyte differentiation markers and possible involvement of microphthalmia transcription factor. J Cell Sci.

[52] Wu X., Yang Y., Xiang L., Zhang C. (2021). The fate of melanocyte: mechanisms of cell death in vitiligo. Pigment Cell Melanoma Res.

[53] Maresca V., Roccella M., Roccella F., Camera E., Del Porto G., Passi S. (1997). Increased sensitivity to peroxidative agents as a possible pathogenic factor of melanocyte damage in vitiligo. J Invest Dermatol.

[54] Speeckaert R., Dugardin J., Lambert J., Lapeere H., Verghaehe E., Speeckaert M.M. (2018). Critical appraisal of the oxidative stress pathway in vitiligo: a systematic review and meta-analysis. J Eur Acad Dermatol Venereol.

[55] Zedan H., Abdel-Motaleb A.A., Kassem N.M., Hafeez H.A., Hussein M.R. (2015). Low glutathione peroxidase activity levels in patients with vitiligo. J Cutan Med Surg.

[56] Akoglu G., Emre S., Metin A., Akbas A., Yorulmaz A., Isikoglu A. (2013). Evaluation of total oxidant and antioxidant status in localized and generalized vitiligo. Clin Exp Dermatol.

[57] Turkcu U.O., Tekin N.S., Edgunlu T.G., Celik S.K., Oner S. (2014). The association of FOXO3A gene polymorphisms with serum FOXO3A levels and oxidative stress markers in vitiligo patients. Gene.

[58] Karsli N., Akcali C., Ozgoztasi O., Kirtak N., Inaloz S. (2014). Role of oxidative stress in the pathogenesis of vitiligo with special emphasis on the antioxidant action of narrowband ultraviolet B phototherapy. J Int Med Res.

[59] Kang P., Zhang W., Chen X., Yi X., Song P., Chang Y. (2018). TRPM2 mediates mitochondria-dependent apoptosis of melanocytes under oxidative stress. Free Radic Biol Med.

[60] Zhang C.F., Gruber F., Ni C., Mildner M., Koening U., Karner S. (2015). Suppression of autophagy dysregulates the antioxidant response and causes premature senescence of melanocytes. J Invest Dermatol.

[61] He Y., Li S., Zhang W., Dai W., Cui T., Wang G. (2017). Dysregulated autophagy increased melanocyte sensitivity to H2O2-induced oxidative stress in vitiligo. Sci Rep.

[62] Rodrigues M., Ezzedine K., Hamzavi I., Pandya A.G., Harris J.E., Group V.W. (2017). New discoveries in the pathogenesis and classification of vitiligo. J Am Acad Dermatol.

[63] Toosi S., Orlow S.J., Manga P. (2012). Vitiligo-inducing phenols activate the unfolded protein response in melanocytes resulting in upregulation of IL6 and IL8. J Invest Dermatol.

[64] Boorn J.G., Picavet D.I., Swieten P.F., Veen H.A., Konijnenberg D., Veelen P.A. (2011). Skin-depigmenting agent monobenzone induces potent T-cell autoimmunity toward pigmented cells by tyrosinase haptenation and melanosome autophagy. J Invest Dermatol.

[65] Bertolotti A., Boniface K., Vergier B., Mossalayi D., Taieb A., Ezzedine K. (2014). Type I interferon signature in the initiation of the immune response in vitiligo. Pigment Cell Melanoma Res.

[66] Harris JE (2016). Cellular stress and innate inflammation in organ-specific autoimmunity: lessons learned from vitiligo. Immunol Ver.

[67] Yu R., Broady R., Huang Y., Wang Y., Yu J., Gao M. (2012). Transcriptome analysis reveals markers of aberrantly activated innate immunity in vitiligo lesional and non-lesional skin. PLoS One.

[68] Tulic M.K., Cavazza E., Cheli Y., Jacquel A., Luci C., Cardot-Leccia (2019). Innate lymphocyte-induced CXCR3B-mediated melanocyte apoptosis is a potential initiator of T-cell autoreactivity in vitiligo. Nat Commun.

[69] Gholijani N., Yazdani M.R., Dastgheib L. (2020). Predominant role of innate proinflammatory cytokines in vitiligo disease. Arch Dermatol Res.

[70] Kroll T.M., Bommiasamy H., Boissy R.E., Hernandez C., Nickoloff B.J., Mestril R. (2005). 4-Tertiary butyl phenol exposure sensitizes human melanocytes to dendritic cell-mediated killing: relevance to vitiligo. J Invest Dermatol.

[71] Zininga T., Ramatsui L., Shonhai A. (2018). Heat shock proteins as immunomodulants. Molecules.

[72] Jacquemin C., Rambert J., Guillet S., Thiolat D., Boukhedouni N., Doutre M.-S. (2017). Heat shock protein 70 potentiates interferon alpha production by plasmacytoid dendritic cells: relevance for cutaneous lupus and vitiligo pathogenesis. Br J Dermatol.

[73] Mosenson J.A., Zloza A., Klarquist J., Barfuss A.J., Guevara-Patino J.A., Poole I.C. (2012). HSP70i is a critical component of the immune response leading to vitiligo. Pigment Cell Melanoma Res.

[74] Mosenson J.A., Zloza A., Nieland J.D., Garrent-Mayer E., Eby J.M., Huelsmann E.J. (2013). Mutant HSP70 reverses autoimmune depigmentation in vitiligo. Sci Transl Med.

[75] Levandowski C.B., Mailloux C.M., Ferrara T.M., Gowan K., Ben S., Jin Y. (2013). NLRP1 haplotypes associated with vitiligo and autoimmunity increase interleukin-1β processing via the NLRP1 inflammasome. Proc Natl Acad Sci U S A.

[76] Dinarello CA (2011). Interleukin-1 in the pathogenesis and treatment of inflammatory diseases. Blood.

[77] Marie J., Kovacs D., Pain C., Jouary T., Cota C., Vergier B. (2014). Inflammasome activation and vitiligo/nonsegmental vitiligo progression. Br J Dermatol.

[78] Boorn J.G., Konijnenberg D., Dellemijn T.A.M., Veen J.P.W., Bos J.D., Melief C.J.M. (2009). Autoimmune destruction of skin melanocytes by perilesional T cells from vitiligo patients. J Invest Dermatol.

[79] Kroon M.W., Kemp E.H., Wind B.S., Krebbers G., Bos J.D., Gawkrodger D.J. (2013). Melanocyte antigen-specific antibodies cannot be used as markers for recent disease activity in patients with vitiligo. J Eur Acad Dermatol Venereol.

[80] Riding RL, Harris JE (2019). The role of memory CD8 ^+^ T cells in vitiligo. J Immunol.

[81] Wankowicz-Kalinska A., Wijngaard RMJGJ, Tigges B.J., Westerhof W., Ogg G.S., Crundolo V. (2003). Immunopolarization of CD4+ and CD8+ T cells to Type-1-like is associated with melanocyte loss in human vitiligo. Lab Invest.

[82] Palermo B., Campanelli R., Garbelli S., Mantovani S., Lantelme E., Brazzelli V. (2001). Specific cytotoxic T lymphocyte responses against Melan-A/MART1, tyrosinase and gp100 in vitiligo by the use of major histocompatibility complex/peptide tetramers: the role of cellular immunity in the etiopathogenesis of vitiligo. J Invest Dermatol.

[83] Harris J.E., Harris T.H., Weninger W., Wherry E.J., Hunter C.A., Turka L.A. (2012). A mouse model of vitiligo with focused epidermal depigmentation requires IFN-gamma for autoreactive CD8(+) T-cell accumulation in the skin. J Invest Dermatol.

[84] Wang X.X., Wang Q.Q., Wu J.Q., Jiang M., Chen L., Zhang C.F. (2016). Increased expression of CXCR3 and its ligands in patients with vitiligo and CXCL10 as a potential clinical marker for vitiligo. Br J Dermatol.

[85] Rashighi M., Agarwal P., Richmond J.M., Harris T.H., Dresser K., Su M.W. (2014). CXCL10 is critical for the progression and maintenance of depigmentation in a mouse model of vitiligo. Sci Transl Med.

[86] Richmond J.M., Masterjohn E., Chu R., Tedstone J., Youd M.E., Harris J.E. (2017). CXCR3 depleting antibodies prevent and reverse vitiligo in mice. J Invest Dermatol.

[87] Frisoli ML, Essien K, Harris JE (2020). Vitiligo: mechanisms of pathogenesis and treatment. Annu Rev Immunol.

[88] Richmond J.M., Strassner J.P., Zapata Jr L., Garg M., Riding R.L., Refat M.A. (2018). Antibody blockade of IL-15 signaling has the potential to durably reverse vitiligo. Sci Transl Med.

[89] Jacquemin C., Martins C., Lucchese F., Thiolat D., Taieb A., Seneschal J. (2020). NKG2D defines a subset of skin effector memory CD8 T cells with proinflammatory functions in vitiligo. J Invest Dermatol.

[90] Chen X., Guo W., Chang Y., Chen J., Kang P., Yi X. (2019). Oxidative stress-induced IL-15 trans-presentation in keratinocytes contributes to CD8(+) T cells activation via JAK-STAT pathway in vitiligo. Free Radic Biol Med.

[91] Ahmed M.B., Zaraa I., Rekik R., Elbeldi-Ferchiou A., Kourda N., Hmida N.B. (2012). Functional defects of peripheral regulatory T lymphocytes in patients with progressive vitiligo. Pigment Cell Melanoma Res.

[92] Mukhatayev Z., Dellacecca E.R., Cosgrove C., Shivde R., Jaishankar D., Pontarolo-Maag K. (2020). Antigen specificity enhances disease control by Tregs in vitiligo. Front Immunol.

[93] Willemsen M., Post N.F., Uden N.O.P., Narayan V.S., Chielie S., Kemp E.H. (2022). Immunophenotypic analysis reveals differences in circulating immune cells in peripheral blood of segmental and nonsegmental vitiligo patients. J Invest Dermatol.

[94] Dahir AM, Thomsen SF (2018). Comorbidities in vitiligo: comprehensive review. Int J Dermatol.

[95] Failla C.M., Carbone M.L., Fortes C., Pagnanelli G., D’Atri S. (2019). Melanoma and vitiligo: in good company. Int J Mol Sci.

[96] Geel N., Speeckaert R., Mollet I., Schepper S., Wolf J., Tjin E.P.M. (2012). In vivo vitiligo induction and therapy model: double-blind, randomized clinical trial. Pigment Cell Melanoma Res.

[97] Hart P.H., Norval M., Byrne S.N., Rhodes L.E. (2019). Exposure to ultraviolet radiation in the modulation of human diseases. Annu Rev Pathol.

[98] Hariharan V., Klarquist J., Reust M.J., Koshoffer A., Mckee M.D., Boissey R.E. (2010). Monobenzyl ether of hydroquinone and 4-tertiary butyl phenol activate markedly different physiological responses in melanocytes: relevance to skin depigmentation. J Invest Dermatol.

[99] Inoue S., Katayama I., Suzuki T., Tanemura A., Ito S., Abe Y. (2021). Rhododendrol-induced leukoderma update II: Pathophysiology, mechanisms, risk evaluation, and possible mechanism-based treatments in comparison with vitiligo. J Dermatol.

[100] Harris JE (2017). Chemical-induced vitiligo. Dermatol Clin.

[101] Arase N., Tanemura A., Jin H., Nishioka M., Aoyoma Y., Oisio N. (2019). Autoantibodies detected in patients with vitiligo vulgaris but not in those with rhododendrol-induced leukoderma. J Dermatol Sci.

[102] Al’Abadie M.S., Senior H.J., Bleehen S.S., Gawkrodger D.J. (1994). Neuropeptide and neuronal marker studies in vitiligo. Br J Dermatol.

[103] Simons R.E., Zevy D.L., Jafferany M. (2020). Psychodermatology of vitiligo: psychological impact and consequences. Dermatol Ther.

[104] Lazarova R., Hristakieva E., Lazarov N., Shani J. (2000). Vitiligo-related neuropeptides in nerve fibers of the skin. Arch Physiol Biochem.

[105] Falabella R., Barona M.I., Echeverri I.C., Alzate A. (2003). Substance P may play a part during depigmentation in vitiligo. A pilot study. J Eur Acad Dermatol Venereol.

[106] Song H., Fang F., Tomasson G., Arnberg F.K., Mataix-Cols D., Cruz L.F. (2018). Association of stress-related disorders with subsequent autoimmune disease. JAMA.

[107] Craiglow BG, King BA (2015). Tofacitinib citrate for the treatment of vitiligo: a pathogenesis-directed therapy. JAMA Dermatol.

[108] Agrawal D., Shajil E.M., Marfatia Y.S., Begum R. (2004). Study on the antioxidant status of vitiligo patients of different age groups in Baroda. Pigment Cell Res.

[109] Vaish U., Kumar A.A., Varshney S., Ghosh S., Sengupta S., Sood C. (2019). Micro RNAs upregulated in Vitiligo skin play an important role in its aetiopathogenesis by altering TRP1 expression and keratinocyte-melanocytes cross-talk. Sci Rep.

[110] Wong P.M., Yang Lil, Yang Lin, Wu H., Li W., Ma X. (2020). New insight into the role of exosomes in vitiligo. Autoimmun Ver.

[111] Bzioueche H., Sjodin K.S., West C.E., Khemis A., Rocchi S., Passeron T. (2021). Analysis of matched skin and gut microbiome of patients with vitiligo reveals deep skin dysbiosis: link with mitochondrial and immune changes. J Invest Dermatol.

[112] Varikasuvu S.R., Aloori S., Varshney S., Bhongir A.V. (2021). Decreased circulatory levels of Vitamin D in Vitiligo: a meta-analysis. An Bras Dermatol.

